# LIM Kinases: From Molecular to Pathological Features

**DOI:** 10.3390/cells12121649

**Published:** 2023-06-16

**Authors:** Hélène Bénédetti, Béatrice Vallée

**Affiliations:** Centre de Biophysique Moléculaire, UPR4301, CNRS, University of Orleans and INSERM, CEDEX 2, 45071 Orleans, France; helene.benedetti@cnrs-orleans.fr

LIM kinases (LIMKs), LIMK1 and LIMK2, are atypical kinases, as they are the only two members of the LIM kinase family harbouring two LIM domains at their N-terminus and a kinase domain at their C-terminus. They are dual kinases able to phosphorylate serine, threonine and tyrosine due to their non-canonical catalytic site. They play a crucial role in cytoskeleton remodelling through the independent regulation of the actin filament and microtubule turnover. Initially, LIMKs were described as downstream effectors of the signalling pathway controlled by small Rho GTPases. Along this pathway, LIMKs phosphorylate cofilin, an actin-depolymerizing factor, leading to its inhibition and actin filament stabilization. The molecular actors of LIMK implication into microtubule dynamics remain unknown. Therefore, many studies on LIMKs have focused on their role involved in cell division, differentiation and migration as aspects of cytoskeleton remodelling.

However, many partners of LIMKs have been identified over the last years, positioning them at the heart of an impressive network of signalling pathways. Along this line, the implications of LIMKs in numerous pathologies have inspired interest in their role as potential relevant therapeutic targets. Many small molecule inhibitors targeting their kinase active site have been developed without success during the clinical stage. As such, it appears crucial to have a better understanding of these kinases to develop new efficient therapies. This Special Issue focuses on these different aspects of LIMKs, presenting an interesting overview of this field and opening new exciting perspectives.

Villalonga et al. [1] propose a broad overview of the LIMK gene and protein organization, as well as their implication in many physiological and pathological processes. They also depicted LIMK partners, substrates and regulators, establishing a detailed scheme of their molecular interactome.

Berabez et al. [2] focus on small molecule inhibitors targeting LIMK kinase activity, and discuss their chemical structure–biological activity relationship. They concentrate on inhibitors that have successfully reached the stage of preclinical assays on animal models of different diseases. They emphasize the failure of these compounds to reach the clinical stage.

Chatterjee et al. [3] highlight the structural features of LIMKs, describing their conformational changes due to ligand binding. They underscore the atypical catalytic mechanism of these kinases, with a focus on substrate recognition and LIMK regulation. They also propose new therapeutic strategies to target these kinases without restricting the field of investigation to their kinase activity.

Ribba et al. [4] shed light on the less known role of LIMKs in embryonic development. They depicted LIMK tissue expression during development, and utilize studies on animal models with loss- or gain-of function mutations in LIMKs and the inhibitors targeting LIMKs in order to better understand their functions.

Park et al. [5] focus on a recently described function of LIMKs in male urogenital system. Indeed, these kinases are involved in several processes crucial for proper urogenital function, such as smooth muscle contraction and spermatogenesis, but also with roles in pathological phenomena, such as cavernosal fibrosis and erectile dysfunction. Therefore, LIMKs may be new therapeutic targets against different urogenital disorders.

Brion et al. [6] draw attention to the implication of LIMKs in osteosarcoma, a bone cancer mainly affecting children and adolescents. Many patients still die from this disease and the survival rates have increased minimally over the last decades; find new therapies against this cancer is an emergency. LIMKs may be one of these new therapeutic targets, as many recent papers described their implication in osteosarcoma. These data are well depicted in this review.

It would also have been interesting to focus on other characteristics of these LIMKs; however, these topics will be briefly mentioned, as limited relevant information has been collated in this Special Issue.

LIMK regulation by miRNA is a well-documented topic in the literature. More than 30 miRNAs targeting LIMKs have been described to regulate their expression, leading to their downregulation and playing a role in various diseases such as cancers, neural pathologies, etc. Long non-coding RNA (LncRNA) and circRNA have also been shown to regulate LIMKs.

Recently, LIMKs have been shown to play a role in viral infections of HIV, Herpes Simplex, Ebola, Rift Valley fiver and Venezuelan Equine Encephalitis viruses, and more studies on this topic would be fruitful [7,8,9].

Furthermore, many papers discuss computational calculations, virtual screening, molecular docking and dynamic simulations, and comparative analysis of different kind of inhibitors (type I, II or II) in order to improve the approach concerning the development of small molecule inhibitors targeting LIMKs [10,11,12,13]. Many efforts are dedicated to this field to improve the pharmacological properties of these molecules and reflect the emergency of finding new compounds in order to selectively target these kinases, with the ultimate goal to reach clinical stages.

The field of research concerning the LIM kinases is still widely open, and many discoveries remain to be achieved to gain a better understanding of these multitasking kinases. We hope you will have interest and enthusiasm to read these well-documented reviews, and that you will widely increase your knowledge on these amazing LIMKs!
